# Supplementary material for: Estimating multiple latencies in the auditory system from auditory steady-state responses on a single EEG channel
Source: Sci Rep. 2021 Jan 25;11:2150. doi: 10.1038/s41598-021-81232-5 (PMC7835249; doi:10.1038/s41598-021-81232-5)
Supplement: Supplementary file 1 — Supplementary Information. [file 41598_2021_81232_MOESM1_ESM.pdf]

Estimating Multiple Latencies in the Auditory System from Auditory Steady-State Responses on a Single EEG Channel - Supplemental Material

Lei Wang, Elisabeth Noordanus, and A. John van Opstal

In this supplemental material, we provide additional details on the phase compensation procedure used in the MSPC method (Appendix A); the simulation results of example 1 in the manuscript (see the main text) under different SNRs (Appendix B); the nonlinear distortions (frequencies and phases) for cosine and sine stimuli (Appendix C); the detailed SNR results of all ASSRs from all subjects (Appendix D); finally, the four stimuli and the resulting second-order and higher-order distortions in this study (Appendix E).

Appendix A
Phase compensation

For linear systems, the output frequencies are exactly the same as the input frequencies, as well as the initial phases (apart from the input-output delay) of the output frequencies. However, this property does not hold if the system is nonlinear. When a nonlinear system is subject to a mixture of I inputs, the output frequencies generated by the rth-order system nonlinearity consist of all possible combinations of the I input frequencies, $\{-f_I, \ldots, -f_1, f_1, \ldots, f_I\}$ taken r items at a time. This property can be described as a set given by:

$$\left\{ f_\Sigma \middle| \begin{array}{c} f_\Sigma = |f_{i_1}+, \ldots, +f_{i_r}| \\ f_i \in \{-f_I, \ldots, -f_1, f_1, \ldots, f_I\}, \; r = 1, \ldots, R \end{array} \right\} \tag{A1}$$

The corresponding initial phases of f_Σ are $\phi(f_\Sigma) = \mathrm{mod}(\frac{\phi(f_{i_1})+,\ldots,+\phi(f_{i_r})}{2\pi})$, where $\phi(\cdot)$ computes the initial phase of a frequency by FT. In particular, for a given r^{th}-order system, the initial phases of the output frequency components are a set:

$$\left\{ \phi(f_{\Sigma_r}) \middle| \begin{array}{c} \phi(f_{\Sigma_r}) = \sum\limits_{i=1}^{r} a_i \phi(f_i), \; with \; \sum\limits_{i=1}^{r} |a_i| = r \\ f_i \in \{f_1, f_2, \ldots, f_I\}, \end{array} \right\} \tag{A2}$$

where a_i is a positive or negative integer. The resulting phase set $\phi(f_{\Sigma_r})$ can be further mapped into the range of [0, 2π] by using the $\mathrm{mod}(\cdot)$ function. We denote the above process as 'phase compensation'. Phase compensation has been used to compute the phase coupling strength between inputs and outputs of nonlinear systems. However, phase compensation is not needed for stimuli when the initial phases of multi-cosine inputs are set to zero (i.e., $\phi(f_i) \equiv 0$). See Appendix C, for further details.

Appendix B
Simulation results of example 1

The SNR in the first column of Table A1 shows the amount of additive Gaussian noise. The SNR in the second column is computed from Eq. (10). The MPE shows the average phase errors (in the 3rd column) of the output frequency components between pure signals and signals with added noise. We simulated 100 trials of 12 sec input signals for each SNR.

TABLE A1
Simulation results with different SNRs

SNR (dB)	SNR*(dB) (m, ±sd)	MPE [0 - 2] (m, ±sd)	LCI (m, ±sd)	Latency (m, ±sd)		Acc (%)**	
				y_1	y_2	y_1	y_2
5	31.2 (±0.33)	0.02 (±0.01)	1.00 (±0.00)	51.0 (±0.04)	21.0 (±0.04)	100	100
0	26.3 (±0.40)	0.03 (±0.01)	0.99 (±0.01)	51.0 (±0.09)	24.8 (±18.8)	100	94
-5	21.2 (±0.36)	0.06 (±0.01)	0.96 (±0.03)	50.9 (±0.2)	38.2 (±41.8)	100	82
-10	16.2 (±0.56)	0.10 (±0.03)	0.86 (±0.10)	51.7 (±8.5)	49.9 (±49.3)	99	65
-15	11.4 (±0.74)	0.19 (±0.04)	0.68 (±0.14)	53.0 (±17.9)	67.8 (±54.2)	92	32
-20	6.6 (±0.98)	0.33 (±0.08)	0.44 (±0.13)	58.6 (±28.0)	71.8 (±50.2)	85	15

* Neighboring SNR: computed from neighboring frequency bins (± 0.5 Hz).
** Accuracy: an estimated latency with an error (± 5 ms) was counted as a correct estimation.

Appendix C
Nonlinear distortions for cosines and sines

Here, we analyze the general properties of the phase shifts of high-order (odd and even-power) nonlinear distortion products for cosine vs. sine inputs. The general power relations for cosine and sine functions are given in (A3) (1st row for odd powers, 2nd row for even powers):

$$\begin{aligned}
&\cos^n\alpha = \tfrac{2}{2^n}\sum_{k=0}^{(n-1)/2}\begin{pmatrix} n \\ k \end{pmatrix}\cos\left((n-2k)\alpha\right) &&\sin^n\alpha = \tfrac{2}{2^n}\sum_{k=0}^{(n-1)/2}(-1)^{\left(\frac{n-1}{2}-k\right)}\begin{pmatrix} n \\ k \end{pmatrix}\sin\left((n-2k)\alpha\right)\\
&\cos^n\alpha = \tfrac{1}{2^n}\begin{pmatrix} n \\ \frac{n}{2} \end{pmatrix}+\tfrac{2}{2^n}\sum_{k=0}^{\frac{n}{2}-1}\begin{pmatrix} n \\ k \end{pmatrix}\cos\left((n-2k)\alpha\right) &&\sin^n\alpha = \tfrac{1}{2^n}\begin{pmatrix} n \\ \frac{n}{2} \end{pmatrix}+\tfrac{2}{2^n}\sum_{k=0}^{\frac{n}{2}-1}(-1)^{\left(\frac{n-1}{2}-k\right)}\begin{pmatrix} n \\ k \end{pmatrix}\cos\left((n-2k)\alpha\right)
\end{aligned} \tag{A3}$$

and together with the multinomial theorem:

$$(x_1+\ldots+x_m)^n = \sum_{k_1+\ldots+k_m=n}\frac{n!}{k_1!k_2!\ldots k_m!}x_1^{k_1}x_2^{k_2}\ldots x_m^{k_m} \tag{A4}$$

a general sum of sines and cosines can be readily computed. As three illustrative examples, we here calculate all phases for the n=2nd, n=3rd and 4th-order powers for a sum of m=3 sines and cosines:

1) Second order: $(x+y+z)^2 = x^2+y^2+z^2+2xy+2xz+2yx$. Applying this to three sine inputs $(\sin\alpha+\sin\beta+\sin\gamma)^2$ yields the following 10 terms:

$$\begin{aligned}
&\frac{3}{2}-\frac{1}{2}\left(\cos(2\alpha)+\cos(2\beta)+\cos(2\gamma)\right)-\frac{1}{2}\left(\cos(\alpha+\beta)+\cos(\alpha+\gamma)+\cos(\beta+\gamma)\right)\\
&+\frac{1}{2}\left(\cos(\alpha-\beta)+\cos(\alpha-\gamma)+\cos(\beta-\gamma)\right)
\end{aligned}$$

If α, α, γ have zero initial phase, the three low-frequency difference distortion components in the output have the same initial phase of $\pi/2$ rad (caused by the transform from sine to cosine). According to (3), this would not affect the estimate from TC, as it will add only a bias to the phase-lag vs. frequency relation, without affecting the slope (latency). However, the six terms following the d.c. all have an initial phase of $3\pi/2$ (due to the negative sign). Thus, if all nine components would be lumped into one set, TC is no longer applicable, because of the different initial phases (and associated biases). Then, phase compensation is required to account for the initial phases of the components. In our EEG example, we employed this property of sines to the second-order difference distortion products in the ASSR.

Whereas three cosine inputs $(\cos\alpha+\cos\beta+\cos\gamma)^2$ yield the following 10 terms:

$$\begin{aligned}
&\frac{3}{2}+\frac{1}{2}\left(\cos(2\alpha)+\cos(2\beta)+\cos(2\gamma)\right)+\frac{1}{2}\left(\cos(\alpha+\beta)+\cos(\alpha+\gamma)+\cos(\beta+\gamma)\right)\\
&+\frac{1}{2}\left(\cos(\alpha-\beta)+\cos(\alpha-\gamma)+\cos(\beta-\gamma)\right)
\end{aligned}$$

Each of the nine distortion components now has the same initial phase of zero rad if α, α, γ have phase zero. Thus, TC is readily applicable to the whole set of distortion components.

2) Third order: $(x+y+z)^3 = x^3+y^3+z^3+6xyz+3x^2y+3x^2z+3y^2x+3y^2z+3z^2x+3z^2y$. The ten terms on the right-hand side contain three third-order powers, one tri-linear product, and six quadratic-linear products, for which the following properties hold.

For sines:

$$\begin{aligned}
\sin^3\theta &= \frac{3}{4}\sin\theta-\frac{1}{4}\sin 3\theta\\
\sin^2\alpha\sin\beta &= \frac{1}{2}\sin\beta-\frac{1}{4}\left[\sin(\beta+2\alpha)+\sin(\beta-2\alpha)\right]\\
\sin\alpha\sin\beta\sin\gamma &= \frac{1}{4}\sin(\alpha+\beta-\gamma)+\frac{1}{4}\sin(\alpha-\beta+\gamma)\\
&\quad-\frac{1}{4}\sin(\alpha+\beta+\gamma)-\frac{1}{4}\sin(\alpha-\beta-\gamma)
\end{aligned}$$

This yields the following 22 terms, with 3 linear components, and 19 distortion products:

$$\frac{7}{4}\left(\sin\alpha+\sin\beta+\sin\gamma\right)-\frac{1}{4}\left(\sin 3\alpha+\sin 3\beta+\sin 3\gamma\right)$$
$$+\frac{3}{2}\left[\begin{array}{l}\sin\left(\alpha+\beta-\gamma\right)+\sin\left(\alpha-\beta+\gamma\right)\\ -\sin\left(\alpha+\beta+\gamma\right)-\sin\left(\alpha-\beta-\gamma\right)\end{array}\right]$$
$$-\frac{3}{4}\left[\begin{array}{l}\sin\left(\alpha+2\beta\right)+\sin\left(\alpha-2\beta\right)+\sin\left(\alpha+2\gamma\right)\\ +\sin\left(\alpha-2\gamma\right)+\sin\left(\beta+2\alpha\right)+\sin\left(\beta-2\alpha\right)\\ +\sin\left(\beta+2\gamma\right)+\sin\left(\beta-2\gamma\right)+\sin\left(\gamma+2\alpha\right)\\ +\sin\left(\gamma-2\alpha\right)+\sin\left(\gamma+2\beta\right)+\sin\left(\gamma-2\beta\right)\end{array}\right]$$

Only the three positive components will have zero phase if α, α, γ have zero phase, but all 19 negative terms will have a phase of π radians. Therefore, the components cannot all be lumped in the phase analysis.

For cosines:

$$\cos^3\theta=\frac{3}{4}\cos\theta+\frac{1}{4}\cos 3\theta$$
$$\cos^2\alpha\cos\beta=\frac{1}{2}\cos\beta+\frac{1}{4}\left[\cos\left(2\alpha+\beta\right)+\cos\left(2\alpha-\beta\right)\right]$$
$$\cos\alpha\cos\beta\cos\gamma=\frac{1}{4}\left[\begin{array}{l}\cos\left(\alpha-\beta+\gamma\right)+\cos\left(\alpha+\beta-\gamma\right)\\ \cos\left(\alpha+\beta+\gamma\right)+\cos\left(\alpha-\beta-\gamma\right)\end{array}\right]$$

Each of the resulting 19 distortion components will maintain their zero phase.

3) Fourth-order nonlinearity: $(x+y+z)^4=x^4+y^4+z^4+4\left(x^3y+x^3z+y^3x+y^3z+z^3x+z^3y\right)+6\left(x^2y^2+x^2z^2+y^2z^2\right)+12(x^2yz+y^2xz+z^2xy)$. The 15 terms on the right-hand side contain three fourth-order powers, six third-order-linear products, three quadratic-quadratic products, and three quadratic-linear-linear products, for which the following properties hold.

For sines:

$$\sin^4\theta=\frac{3}{8}-\frac{1}{2}\cos 2\theta+\frac{1}{8}\cos 4\theta$$
$$\sin^3\alpha\sin\beta=\frac{3}{8}\left[\cos\left(\alpha-\beta\right)-\cos\left(\alpha+\beta\right)\right]-\frac{1}{8}\left[\cos\left(3\alpha-\beta\right)-\cos\left(3\alpha+\beta\right)\right]$$
$$\sin^2\alpha\sin^2\beta=\frac{1}{4}\left[\begin{array}{l}1-\cos 2\alpha-\cos 2\beta+\\ \frac{1}{2}\cos\left(2\alpha+2\beta\right)+\frac{1}{2}\cos\left(2\alpha-2\beta\right)\end{array}\right]$$
$$\cos^2\alpha\cos\beta\cos\gamma=\frac{1}{4}\left[\cos\left(\beta-\gamma\right)+\cos\left(\beta+\gamma\right)\right]+\frac{1}{8}\left[\cos\left(2\alpha-\beta-\gamma\right)+\cos\left(2\alpha+\beta+\gamma\right)\right]+\frac{1}{8}\left[\cos\left(2\alpha-\beta+\gamma\right)+\cos\left(2\alpha+\beta-\gamma\right)\right]$$

Now, all terms will yield phase-shifted cosines of either $\pi/2$ (positive terms) or $3\pi/2$ (negative terms) at t=0, which means that they will follow different (shifted) phase-frequency relations and cannot be lumped. Note that the three difference-frequency terms will all have the same $\pi/2$ phase shift as produced by the second-order nonlinearity.

For cosines:

$$\cos^4\theta=\frac{3}{8}+\frac{1}{2}\cos 2\theta+\frac{1}{8}\cos 4\theta$$
$$\cos^3\alpha\cos\beta=\frac{3}{8}\left[\cos\left(\alpha-\beta\right)-\cos\left(\alpha+\beta\right)\right]+\frac{1}{8}\left[\cos\left(3\alpha-\beta\right)-\cos\left(3\alpha+\beta\right)\right]$$
$$\cos^2\alpha\cos^2\beta=\frac{1}{4}\left[\begin{array}{l}1+\cos 2\alpha+\cos 2\beta+\\ \frac{1}{2}\cos\left(2\alpha+2\beta\right)+\frac{1}{2}\cos\left(2\alpha-2\beta\right)\end{array}\right]$$
$$\cos^2\alpha\cos\beta\cos\gamma=\frac{1}{4}\left[\cos\left(\beta-\gamma\right)+\cos\left(\beta+\gamma\right)\right]+\frac{1}{8}\left[\cos\left(2\alpha-\beta-\gamma\right)+\cos\left(2\alpha+\beta+\gamma\right)\right]+\frac{1}{8}\left[\cos\left(2\alpha-\beta+\gamma\right)+\cos\left(2\alpha+\beta-\gamma\right)\right]$$

Again, all phases of the 54 different distortion products will remain zero at t=0.

In conclusion: from this analysis and from the general equations (A3) and (A4), it can be readily appreciated that the above properties for sine and cosine inputs will hold, irrespective of the order of the nonlinearity n, and of the number of input frequencies m. We can hence conclude that cosine inputs will always maintain zero phase for all nonlinear distortions, but for sine inputs the situation becomes much more complex because, depending on the contributing nonlinearity, the phases of the distortion components can be either 0, $\pi/2$, π, or $3\pi/2$. However, the phase of the difference frequencies, generated by all even-order nonlinearities, will always be $\pi/2$, and therefore can be unambiguously used in the non-parametric ALPC-SFS method, as has been applied in our EEG data analysis.

Appendix D
SNR results from all subjects.

Figure A1 shows the detailed SNR values on one EEG channel (FCz) for each subject and for the two recording days.

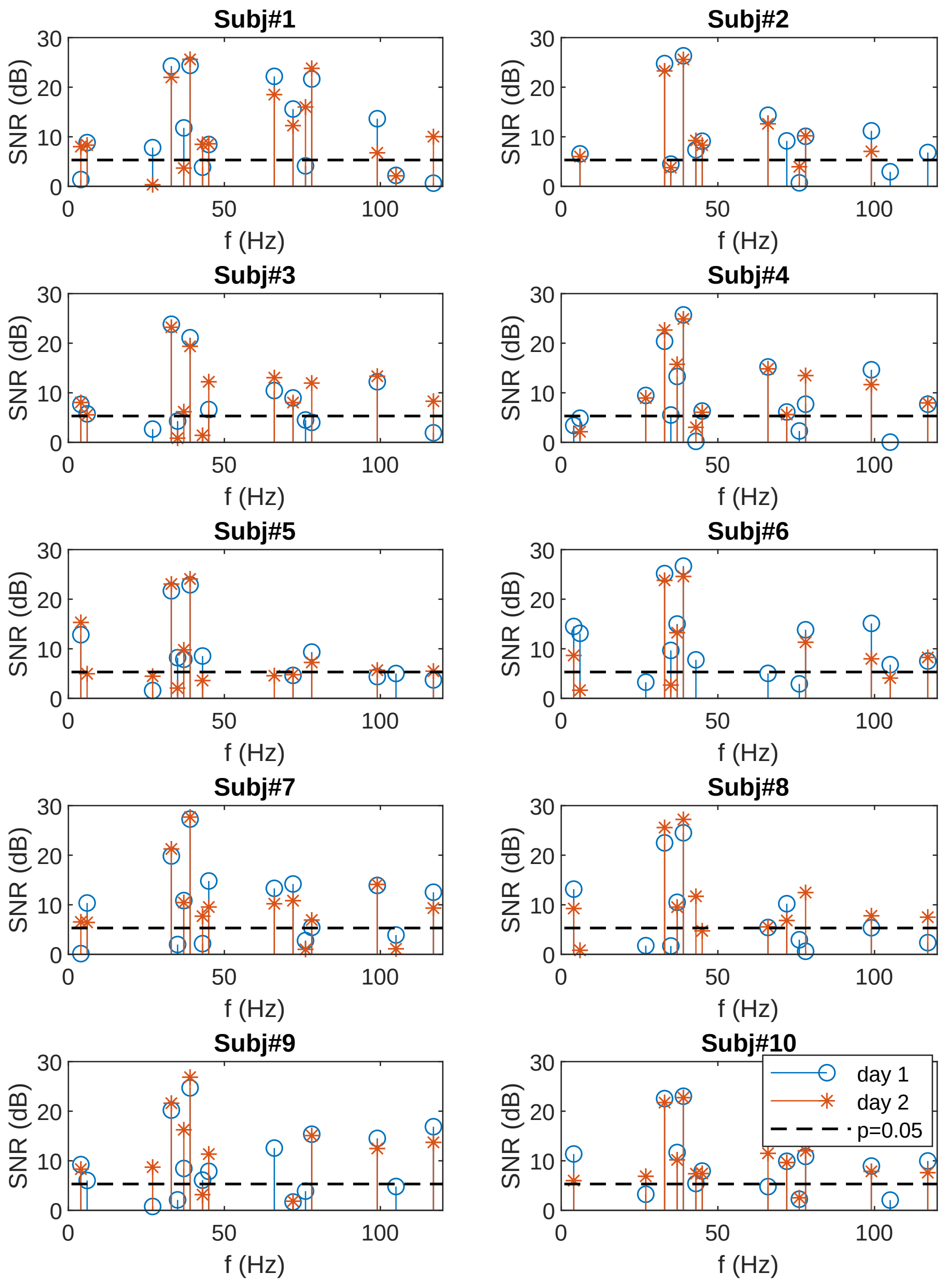

Fig. A1. SNRs on the target ASSR frequencies for the two measurements of 10 subjects (EEG channel FCz as an example here).

APPENDIX E
STIMULI AND THEIR DISTORTION PRODUCTS.

Figure A2 illustrates the four stimuli and the resulting second-order and higher-order distortions.

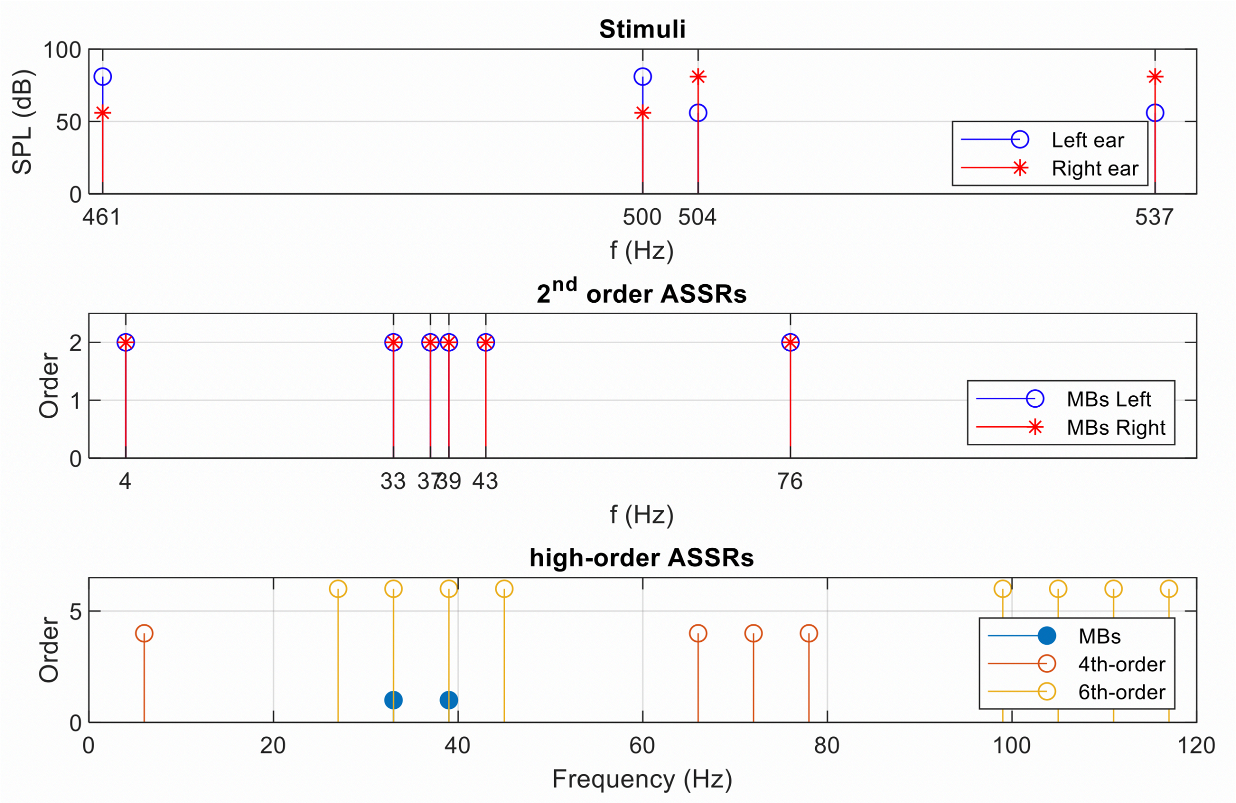

Fig. A2. Stimulus frequencies presented at the left- (blue) and right-ear (red) (scaled according to their intensities), and their second and higher-order (monaural) distortions.
